# Lysosomal Dysfunction in Amyotrophic Lateral Sclerosis: A Familial Case Linked to the p.G376D *TARDBP* Mutation

**DOI:** 10.3390/ijms26072867

**Published:** 2025-03-21

**Authors:** Roberta Romano, Victoria Stefania Del Fiore, Giorgia Ruotolo, Martina Mazzoni, Jessica Rosati, Francesca Luisa Conforti, Cecilia Bucci

**Affiliations:** 1 Department of Experimental Medicine, University of Salento, Via Provinciale Lecce-Monteroni n. 165, 73100 Lecce, Italy; victoriastefania.delfiore@unisalento.it; 2 Cell Reprogramming Unit, Fondazione IRCCS Casa Sollievo della Sofferenza, Viale dei Cappuccini, 71013 San Giovanni Rotondo, Italy; g.ruotolo@operapadrepio.it (G.R.); m.mazzoni@operapadrepio.it (M.M.); j.rosati@operapadrepio.it (J.R.); 3 Department of Biotechnology and Biosciences, University of Milano-Bicocca, 20126 Milan, Italy; 4 Departmental Faculty of Medicine, UniCamillus-Saint Camillus International University of Health Sciences, Via di Sant’Alessandro, 8, 00131 Rome, Italy; 5 Department of Pharmacy, Health and Nutritional Sciences, University of Calabria, 87036 Rende, Italy; francescaluisa.conforti@unical.it

**Keywords:** neurodegeneration, neurodegenerative disease, lysosome, TDP-43, TFEB, amyotrophic lateral sclerosis

## Abstract

Amyotrophic lateral sclerosis (ALS) is a fatal neurodegenerative disease affecting motor neurons. Consequent to the loss of these cells, neuromuscular functions decline, causing progressive weakness, muscle wasting, and paralysis, leading to death in 2 to 5 years. More than 90% of ALS cases are sporadic, while the remaining 10% of cases are familial, due to mutations in 40 different genes. One of the most common genes to be mutated in ALS is *TARDBP* (transactive response DNA binding protein 43), which encodes TDP-43 (TAR DNA-binding protein 43). A mutation in exon 6 of *TARDBP* causes the aminoacidic substitution G376D in the C-terminal region of TDP-43, leading to its cytoplasmic mislocalization and aggregation. In fibroblasts derived from patients carrying this mutation, we found a strong increase in lysosome number, with overexpression and higher nuclear translocation of the transcription factor TFEB. In contrast, lysosomal functionality was deeply compromised. Interestingly, lysosomal activity was unaffected at an early stage of the disease, worsening in more advanced stages. Moreover, we observed the same pathological phenotype in iPSC (induced pluripotent stem cells)-derived patient motor neurons carrying the G376D mutation. Therefore, this mutation compromises the functionality of lysosomes, possibly contributing to neurodegeneration.

## 1. Introduction

Amyotrophic lateral sclerosis is a neurodegenerative disorder caused by the degeneration of both upper and lower motor neurons [1,2]. Consequent to the loss of these cells, neuromuscular functions decline and cause progressive weakness, muscle wasting, and paralysis, leading to death in 2–5 years because of respiratory complications due to respiratory muscle atrophy [3,4]. More than 90% of ALS cases are sporadic (sALS), while the remaining 10% of cases are familial (fALS), and are due to mutations in about 40 different genes. The most common mutations have been identified in *SOD1* (superoxide dismutase 1), *C9orf72* (chromosome 9 open reading frame 72), *TARDBP* (transactive response DNA binding protein 43), and *FUS* (fused in sarcoma) [5]. Apart from *SOD1* and *C9orf72*, the most common gene to be mutated in ALS is *TARDBP,* which encodes TDP-43 (TAR DNA-binding protein 43) [5].

sALS and fALS cannot be distinguished from the clinical point of view, and are both characterized by intracellular protein aggregates. In particular, ubiquitinated and hyper-phosphorylated cytosolic aggregates in ALS are composed mainly of TDP-43. These cytoplasmic inclusions represent a hallmark of ALS, as they appear in 97% of cases, regardless of the mechanisms of disease onset. More than 50 mutations in *TARDPB* are associated with ALS onset, making the alterations of TDP-43 functions critical for ALS [6].

TDP-43 is 414 amino acids long, and its structure comprises an N-terminal region with a nuclear localization signal (NLS), two RNA recognition motifs (RRMs), a nuclear export signal (NES), and a C-terminal region characterized by a prion-like glutamine/asparagine-rich domain and a region rich in glycine [3,7]. The localization of TDP-43 is predominantly in the nucleus, but it can shuttle to the cytoplasm for some of its functions [8,9].

The pathological behavior of TDP-43 seems to be linked to the C-terminal region for several reasons. It is intrinsically prone to aggregation [10], and most ALS-associated mutations have been found in this region [11]. The aberrant activity of caspases produces highly cytotoxic C-terminal fragments of 25–35 kDa, accumulating in the inclusion bodies [11]. An unstable helix–turn–helix region can also be found in this region, from which peptides can originate and form amyloid-like fibrils in vitro [12,13].

TDP-43 participates in RNA metabolism, regulating transcription, translation, mRNA transport and stabilization, and the processing of microRNA and long non-coding RNA [14,15]. Importantly, genome-wide RNA immunoprecipitation techniques have shown that TDP-43 is associated with more than 6000 mRNAs, representing 30% of the entire transcriptome [16,17,18]. Among them, there are mRNAs involved in the regulation of autophagy, such as ATG7 (autophagy-related 7) [19]. Moreover, the localization of TFEB (transcription factor EB), a transcription factor critical for lysosomal function and biogenesis, is affected by TDP-43 [20]. Indeed, TDP-43 silencing increases the nuclear localization of this transcription factor, leading to alterations in the expression levels of several autophagy and lysosomal proteins and a reduced autophagic flux [20]. Therefore, lysosomal function is maintained by TDP-43, which regulates the expression and the intracellular localization of several genes that are essential for the proper activity of the autophagy and lysosome pathways [21].

A mutation in exon 6 of the *TARDBP* gene (c. 1127 G > A) was detected in an Italian family with several members affected by ALS [22]. This mutation determines an aminoacidic substitution in the C-terminal region of the protein (p.G376D), and it is associated with the mislocalization of TDP-43, cytoplasmic aggregation, and reduced protein amount in the nucleus [22,23].

Considering that TDP-43 is involved in the regulation of lysosomal function, and that the G376D mutation in TDP-43 determines the mislocalization of the protein, affecting its nuclear functions, we analyzed the effect of this mutation on lysosomes in patient fibroblasts and iPSC (induced pluripotent stem cell)-derived motor neurons. We demonstrated that ALS cells have more lysosomes as a consequence of an increase in TFEB abundance and nuclear translocation. However, lysosomal functionality is deeply compromised in patients’ cells. Therefore, this study reveals that the G376D mutation in TDP-43 affects lysosomal activity, disrupting cell homeostasis and contributing to neurodegeneration.

## 2. Results

### 2.1. Lysosomal Functionality Is Altered in Cells Expressing the TDP-43^G376D^ Mutant Protein

In a previous study, we demonstrated that the TDP-43^G376D^ mutation caused the formation of TDP-43 cytoplasmic aggregates, possibly affecting TDP-43 nuclear functions [24]. As TDP-43 regulates several genes involved in the lysosomal pathway, we decided to analyze whether patient cells carrying the TDP-43^G376D^ mutation show lysosomal alterations. As cellular models, we used dermal fibroblasts obtained from two healthy controls (CTRL1 and 2) and from two ALS patients carrying the TDP-43^G376D^ mutation who were at an advanced stage of the disease (called ALS1A and ALS2A) [24]. In addition, ALS1O fibroblasts were obtained from patient 1 at the time of the diagnosis [24].

**Figure 1:**
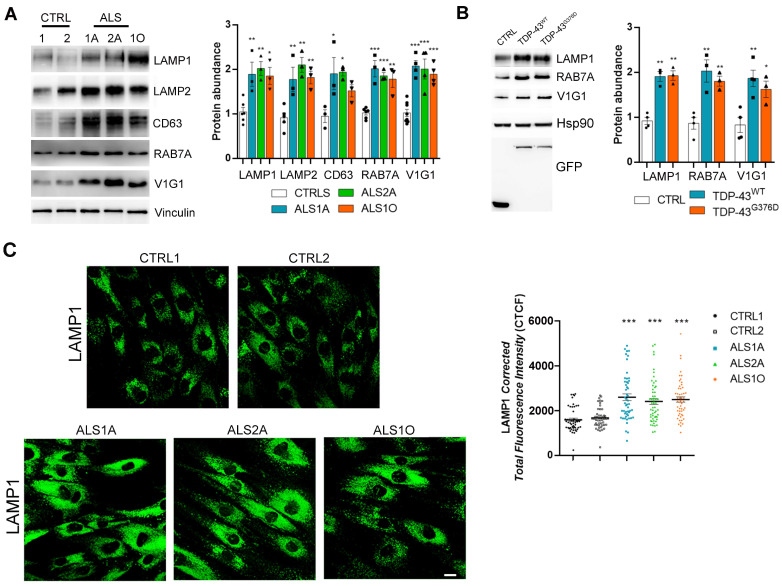
TDP-43^G376D^ is associated with increased expression of late endocytic and lysosomal markers. (**A**,**B**) Lysates of control and ALS fibroblasts (**A**), and of HeLa cells expressing GFP, GFP-TDP-43 wild-type, or G376D, as indicated (**B**), were analyzed by Western blot; antibodies against LAMP1, LAMP2, CD63, RAB7A, V1G1, vinculin, Hsp90 and GFP were used. Vinculin or Hsp90 was used as loading controls, and GFP was used to check transfection. Statistical analysis was performed using one-way ANOVA, followed by Dunnett’s test for multiple comparisons. * = *p* < 0.05; ** = *p* < 0.01; *** = *p* < 0.001. (**C**) Control and ALS fibroblasts were immunolabeled with anti-LAMP1, followed by Alexa488-conjugated secondary antibody. Bar = 10 µm. Statistical analysis was performed using one-way ANOVA, followed by Dunnett’s test for multiple comparisons. * = *p* < 0.05; ** = *p* < 0.01; *** = *p* < 0.001.

We first analyzed the expression of several markers involved in the lysosomal pathway by Western blot analysis. In particular, we evaluated the abundance of LAMP (lysosomal-associated membrane protein); LAMP1 and LAMP2, which are routinely used as late endosomal and lysosomal markers [25]; the Tetraspanin CD63/LAMP3, a component of the late endosomal and lysosomal membranes [26]; RAB7A, whose abundance increases during early-to-late endosome transition, and which is also important for autophagosome maturation [27,28]; and V1G1, a subunit of the V (vacuolar)-ATPase essential for the acidification of lysosomal lumen [29,30]. We found that the abundance of all these markers was enhanced in fibroblasts expressing the TDP-43^G376D^ mutant protein compared to control cells, suggesting an increased presence of late endosomes and lysosomes in patients’ cells (Figure 1A).

As we could analyze cells from only two patients, to confirm that the variations observed were caused by the expression of the mutant protein and not by individual variability between control and patient individuals, we transfected HeLa cells with plasmids coding for the TDP-43 wild-type or the G376D mutant protein, and we evaluated the abundance of LAMP1, RAB7A, and V1G1. The exogenous expression of the TDP-43^G376D^ mutant protein in HeLa cells caused increased expression of all three markers, confirming the findings observed in patient cells (Figure 1B).

To determine whether ALS fibroblasts endogenously expressing the TDP-43^G376D^ mutant protein possess more lysosomes, we performed an immunofluorescence analysis using an antibody against LAMP1. We observed increased staining of LAMP-1-positive organelles in cells endogenously expressing the TDP-43^G376D^ mutant protein, compared to control cells (Figure 1C), suggesting the presence of an increased number of lysosomes in these cells.

**Figure 2:**
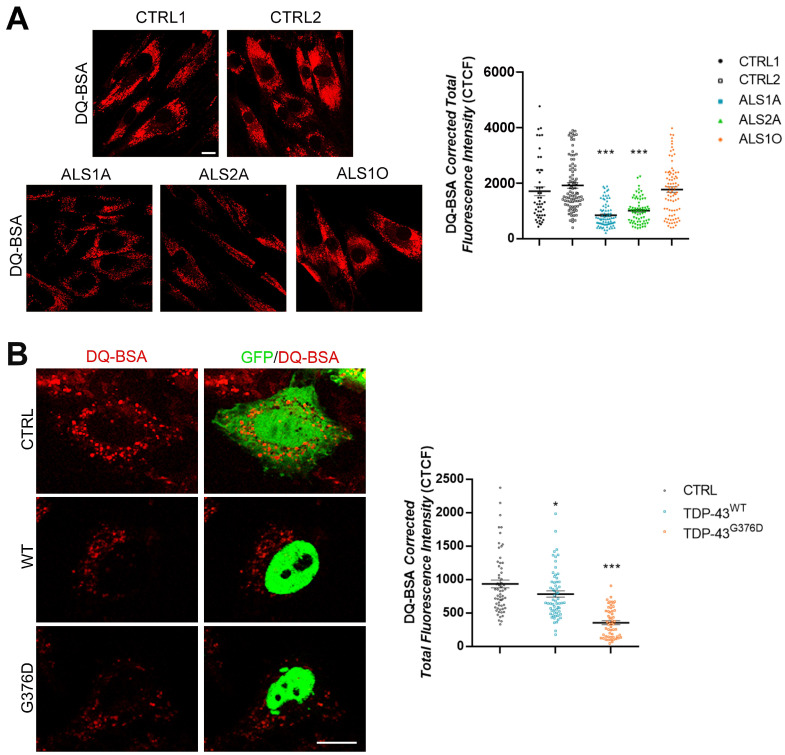
Lysosomes are dysfunctional in ALS cells carrying the TDP-43^G376D^ mutation. (**A**) Control and ALS fibroblasts were treated with 10 µM DQ-BSA for 48 h, and then fixed. (**B**) HeLa cells were transfected with plasmids coding for GFP, GFP-TDP-43 wild-type, or G376D. When it had been 24 h since transfection, they were treated with 25 µM DQ-BSA for 24 h, and then fixed. Bar = 10 µm. Statistical analysis was performed using one-way ANOVA, followed by Dunnett’s test for multiple comparisons. ImageJ software 1.54d was used for the CTCF calculation of at least 50 cells per experiment. * = *p* < 0.05; *** = *p* < 0.001.

Then, we wondered whether lysosomal functionality was altered in these cells. To answer this question, we treated fibroblasts with DQ^TM^ BSA. This molecule is cleaved in degradative acidic compartments, generating highly fluorescent products; therefore, fluorescence quantification estimates lysosomal degradative activity. Fluorescence intensity was lower in ALS1A and ALS2A cells, demonstrating reduced lysosomal activity in these cells compared to control cells (Figure 2A). Notably, ALS1O cells showed a DQ-BSA fluorescence intensity comparable to that of control cells, indicating that lysosomal functionality worsens with the progression of the disease.

To confirm that the reduced fluorescence of DQ-BSA observed in ALS fibroblasts was due to mutant TDP-43, we transfected HeLa cells with plasmids encoding GFP (as control), and wild-type or mutant TDP-43. Interestingly, the expression of both wild-type and mutant proteins led to a decreased fluorescence of DQ-BSA, which was particularly significant in the case of the mutant protein (Figure 2B).

**Figure 3:**
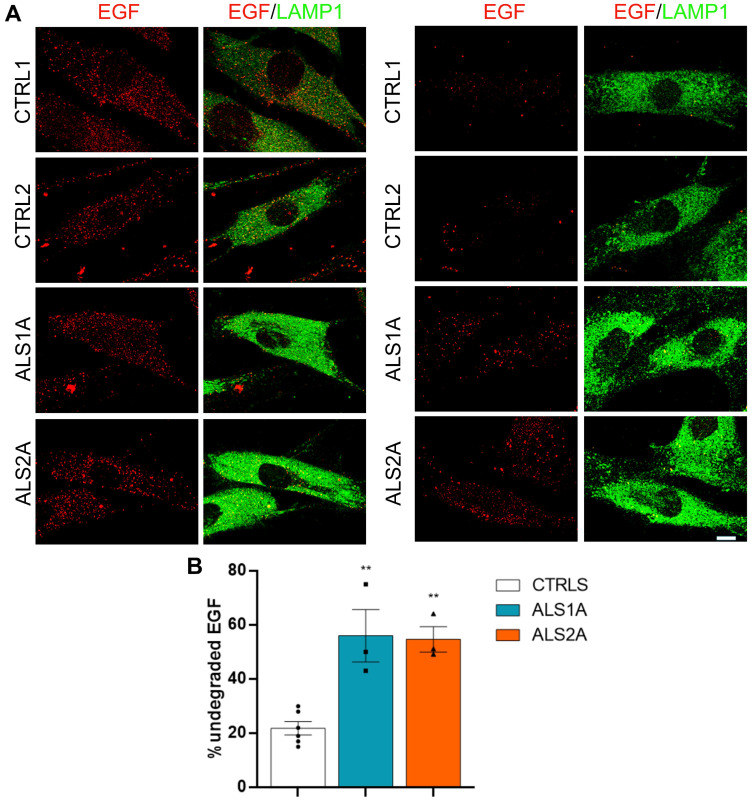
EGF degradation is inhibited in ALS fibroblasts. (**A**) Control and ALS fibroblasts were starved overnight, and then treated with rhodamine-labeled EGF in a starvation medium for 1 h. After several washes, cells were incubated with complete DMEM for 15 min (**left** panels) and 4 h (**right** panels). After these periods, cells were fixed and immunolabeled using an anti-LAMP1 antibody, followed by an Alexa488-conjugated antibody. Bar = 10 µm. (**B**) The CTCF of EGF was quantified and plotted as the percentage of the respective fluorescence intensities after 15 min of incubation with DMEM. Statistical analysis was performed using one-way ANOVA, followed by Dunnett’s test for multiple comparisons. ImageJ software 1.54d was used for the CTCF calculation of at least 50 cells per experiment. ** = *p* < 0.01.

Moreover, we evaluated EGF (epidermal growth factor) degradation to further confirm the impairment of lysosomal activity. We starved control and ALS fibroblasts, and then incubated them with rhodamine-labeled EGF for 1 h. After this period, we incubated cells with complete DMEM for 15 min and 4 h to follow EGF degradation. Interestingly, while in control cells, most of the EGF staining disappeared after 4 h of incubation, indicating that EGF was degraded; in ALS fibroblasts, most of the EGF staining remained, confirming lysosomal dysfunctions (Figure 3A,B).

Altogether, these data indicate that the presence of the TDP-43^G376D^ mutant protein causes an increase in the number of lysosomes, but reduced lysosomal activity, suggesting that the presence of the TDP-43^G376D^ mutant protein negatively influences the lysosomal pathway.

### 2.2. ALS Fibroblasts Display Abnormal Lysosomal Acidification

**Figure 4:**
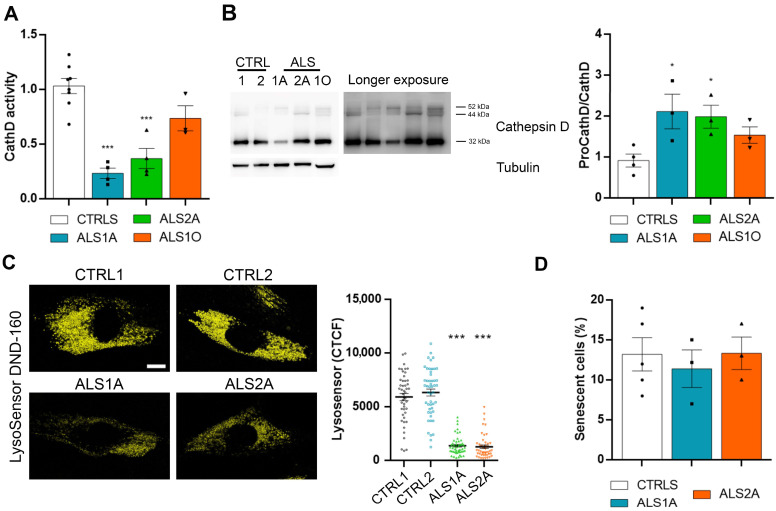
ALS fibroblasts show impaired cathepsin D activity and defective acidification. (**A**) A Cathepsin D activity assay was performed in control and ALS fibroblasts. (**B**) Lysates of control and ALS fibroblasts were subjected to Western blot analysis using anti-cathepsin D and anti-tubulin antibodies. Bar = 10 µm. (**C**) Control and ALS fibroblasts were labeled with Lysosensor DND-160 for 5 min at 37 °C. The CTCF quantification is shown. Bar = 10 µm. (**D**) β-galactosidase staining of control and ALS fibroblasts. Senescent cells were counted and normalized on total cells. Statistical analysis of all panels was performed using one-way ANOVA, followed by Dunnett’s test for multiple comparisons. * = *p* < 0.05; *** = *p* < 0.001.

Cathepsin D is fundamental for cellular homeostasis, being the most abundant protease in lysosomes [31]. To assess its activity in control and ALS fibroblasts, we performed an assay based on fluorescence release after the cleavage of a Cathepsin D substrate. This experiment demonstrated that this protease is less active in ALS fibroblasts. Interestingly, we did not detect significant differences between ALS1O and control fibroblasts (Figure 4A).

Furthermore, Cathepsin D is fundamental for lysosomal functions, but it requires that lysosomes are fully functional for its activation. Indeed, 52 kDa pro-cathepsin D is cleaved into an intermediate form of 44 kDa, and then further processed into the mature form of 32 kDa in lysosomes [31]. We quantified the ratio between immature forms and the 32 kDa mature form using an antibody able to recognize the immature and mature forms. The result showed an increased ratio, indicating impairment of cathepsin D maturation (Figure 4B).

As degradative organelles, lysosomes should have a pH of 4.0–5.0 in their lumen [32]. Therefore, having demonstrated an impairment of the degradative activity in ALS fibroblasts, we evaluated acidification using Lysosensor DND-160. Interestingly, we observed a strong reduction in Lysosensor fluorescence in ALS fibroblasts, demonstrating defective acidification in cells carrying the TDP-43^G376D^ mutation (Figure 4C).

Considering that increased lysosomal mass is a characteristic of senescent cells [33], to exclude the possibility that the phenotypes that we observed in ALS fibroblasts were related to the senescence of cells, and not to the presence of the TDP-43^G376D^ mutation, we performed a senescence-associate β-galactosidase assay. Control and ALS fibroblasts at the same passage were stained using X-gal, and we measured the activity of β-galactosidase. This chromogenic assay did not reveal significant differences between control and ALS fibroblasts, demonstrating that the increased number of lysosomes was due to mutant TDP-43, and not to senescence (Figure 4D).

### 2.3. TFEB Nuclear Translocation Is Increased in Cells Expressing the TDP-43^G376D^ Mutant Protein

**Figure 5:**
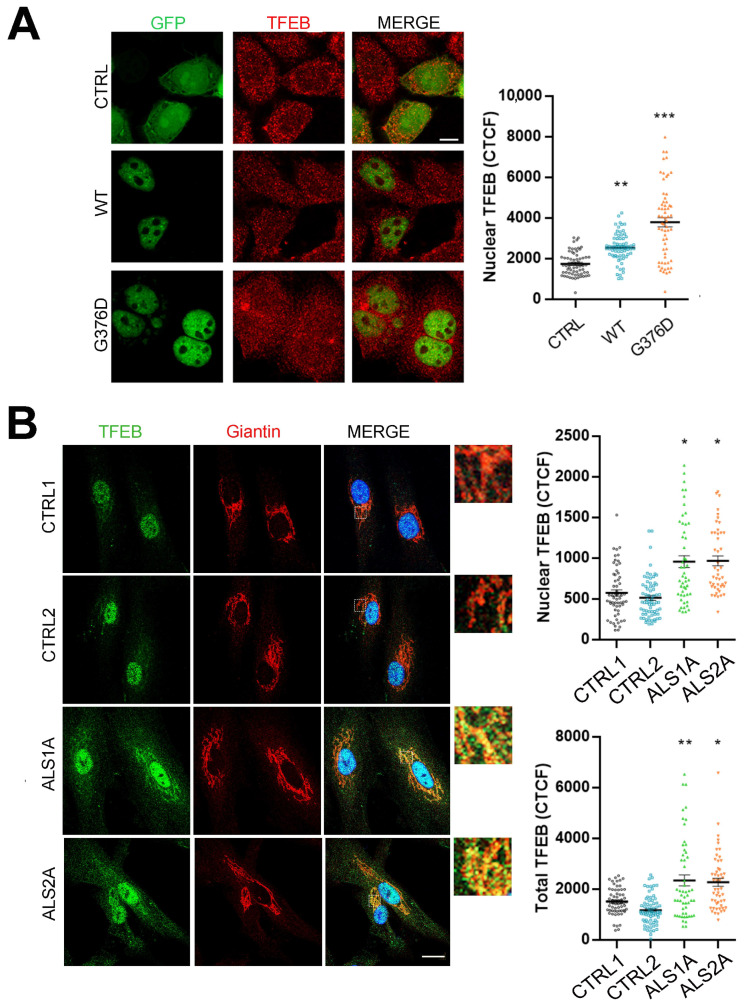
TFEB abundance and nuclear translocation are increased in TDP-43^G376D^-expressing cells. HeLa cells transfected with a plasmid encoding EGFP (CTRL), EGFP-TDP-43 wild-type, or EGFP-TDP-43^G376D^ for 48 h (**A**), or control and ALS fibroblasts (**B**) were immunolabeled with TFEB alone or in combination with Giantin antibody, followed by Alexa488- and Alexa568-conjugated antibodies. Bar = 10 µM. The Corrected Total Cell Fluorescence (CTCF) was calculated, and statistical analysis was performed using one-way ANOVA, followed by Dunnett’s test for multiple comparisons. * = *p* < 0.05; ** = *p* < 0.01; *** = *p* < 0.001.

Considering that TDP-43 silencing induces increased TFEB activity [20], and that we observed a higher abundance of acidic compartments in ALS patient fibroblasts endogenously expressing the TDP-43^G376D^ mutant protein, we wondered whether TFEB mediated the effects observed upon expression of the TDP-43^G376D^ mutant protein. To this purpose, we transfected HeLa cells with plasmids coding for EGFP as a control, and with plasmids coding for EGFP-tagged TDP-43 wild-type or the mutant protein, and we evaluated TFEB nuclear translocation by an immunofluorescence assay. Notably, the nuclear fluorescence intensity of TFEB was higher in cells overexpressing wild-type TDP-43 than in control cells, and it showed an even greater intensity in cells expressing the mutant protein TDP-43^G376D^ (Figure 5A).

We also looked at TFEB intracellular localization in control and ALS fibroblasts endogenously expressing the TDP-43^G376D^ mutant protein to confirm these data. We observed increased nuclear TFEB translocation and a significant increase in the total amount of this transcription factor in ALS cells (Figure 5B). Interestingly, we also detected an accumulation of TFEB in the Golgi apparatus in ALS fibroblasts endogenously expressing the TDP-43^G376D^ mutant protein, as demonstrated by TFEB colocalization with the Golgi marker Giantin (Figure 5B).

**Figure 6:**
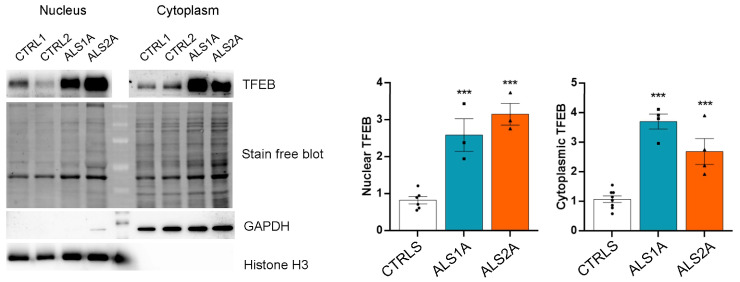
TFEB abundance is increased in ALS cells carrying the TDP-43^G376D^ mutation. Cytoplasmic and nuclear fractions of control and ALS fibroblasts were subjected to Western blotting using anti-TFEB, anti-GAPDH, and anti-histone H3 antibodies. Stain-Free Imaging Technology (BIO-RAD) was used for the loading control. GAPDH and histone H3 were used as cytosolic and nuclear markers, respectively. The quantification of nuclear and cytoplasmic TFEB is shown. Statistical analysis was performed using one-way ANOVA, followed by Dunnett’s test for multiple comparisons. *** = *p* < 0.001.

Then, we decided to separate the nuclear and cytoplasmic fractions of control and patients’ fibroblasts to confirm the data obtained by immunofluorescence analysis, and to quantify the total and nuclear amount of TFEB by Western blot analysis. Notably, TFEB was more abundant in both fractions in ALS fibroblasts, confirming a substantial increase in the total amount of TFEB in the presence of the TDP-43^G376D^ mutant protein (Figure 6). In addition, in fibroblasts expressing the TDP-43^G376D^ mutant protein, we observed a 2.5- and 3-fold increase in nuclear TFEB in ALS1A and ALS2A, respectively, compared to control cells (Figure 6). These data suggest that the more significant activity of this transcription factor in the nucleus could be responsible for the enhanced lysosomal biogenesis observed in these cells.

### 2.4. Increased Nuclear Translocation of TFEB Depends on Reduced Activation of AKT

Previous studies have shown that mTORC1 regulates TFEB activity [34]. Indeed, when mTOR localizes to lysosomes, it phosphorylates TFEB on serine 211, contributing to the retention of TFEB in the cytosol because of its interaction with the 14-3-3 protein [34]. In contrast, when the lysosomal function is deficient, there is reduced phosphorylation of TFEB, impairing the interaction with the 14-3-3 protein and causing TFEB nuclear translocation [34]. Raptor is a fundamental factor for mTOR lysosomal localization, as it targets mTOR to lysosomes [35]. Therefore, decreased mTORC1 lysosomal localization caused by a decrease in Raptor abundance could be responsible for reduced TFEB phosphorylation and, thus, increased translocation of TFEB in the nucleus.

**Figure 7:**
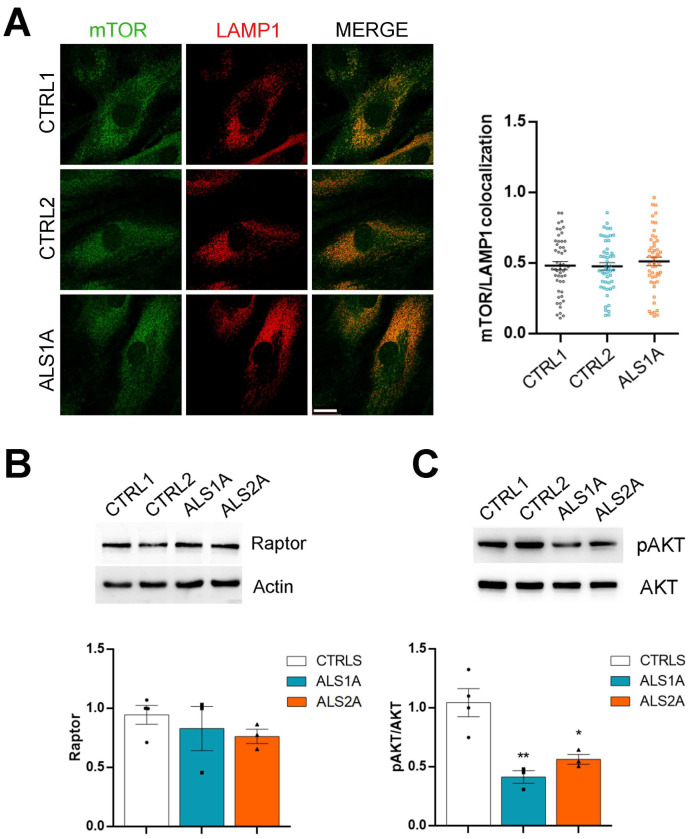
The TDP-43^G376D^ mutation increases TFEB nuclear translocation in an AKT-dependent manner. (**A**) Control and ALS fibroblasts were immunolabeled with mTOR and LAMP1 antibodies, followed by Alexa568- and Alexa488-conjugated antibodies. Bar = 10 µM. Colocalization between mTOR and LAMP1 was quantified. Statistical analysis was performed using one-way ANOVA, followed by Dunnett’s test for multiple comparisons. (**B**,**C**) Control and ALS fibroblasts were analyzed by Western blot, using antibodies against Raptor and β-Actin (**B**), or using antibodies against pAKT and AKT. β-Actin or AKT were used as loading controls. (**C**). Statistical analysis was performed using one-way ANOVA, followed by Dunnett’s test for multiple comparisons. * = *p* < 0.05; ** = *p* < 0.01.

To understand whether the increased TFEB nuclear translocation observed in the presence of the TDP-43^G376D^ mutant protein depends on mTORC1 activity, we evaluated the colocalization between mTOR and LAMP1 by immunofluorescence, and we found no differences in fibroblasts endogenously expressing the TDP-43^G376D^ mutant protein compared to control fibroblasts (Figure 7A).

Moreover, we evaluated the abundance of Raptor by Western blot analysis, and, coherently, Raptor protein levels were comparable between control and ALS fibroblasts (Figure 7B). These data indicate that the increased nuclear translocation of TFEB observed in TDP-43^G376D^-expressing cells is not dependent on reduced phosphorylation from mTORC1 and Raptor.

An alternative mechanism of regulation of TFEB activity is mediated by AKT kinase, in an mTORC1-independent manner [36]. Indeed, AKT phosphorylates TFEB on serine 467, repressing TFEB nuclear translocation, while lower AKT activity favors it [36]. Therefore, we wondered whether the increased TFEB nuclear translocation observed in ALS fibroblasts carrying the TDP43^G376D^ mutation could be due to a reduced amount of AKT or reduced AKT activation in ALS fibroblasts. We evaluated the AKT amount and phosphorylation by Western blot analysis (Figure 7C) to answer this question. We found that the AKT amount was not different in ALS patient fibroblasts compared to controls. However, the phosphorylated form of AKT showed a significant decrease in fibroblasts carrying the TDP-43^G376D^ mutation, compared to control fibroblasts. These data indicate that in ALS fibroblasts, there is reduced activation of AKT, which is responsible for TFEB nuclear translocation and increased lysosomal biogenesis in these cells (Figure 7C).

### 2.5. ALS Motor Neurons Carrying the G376D Mutation in TDP-43 Show Reduced Lysosomal Activity

To confirm some of the data obtained in ALS patient fibroblasts using a more appropriate cellular model, considering that ALS affects motor neurons, we used induced pluripotent stem cells (iPSCs) obtained by reprogramming skin fibroblasts derived from healthy controls and ALS patient 1 at the time of diagnosis and four years after diagnosis (ALS1O and ALS1A, respectively) [37,38]. We differentiated them into motor neurons, as detailed in the Materials and Methods Section.

**Figure 8:**
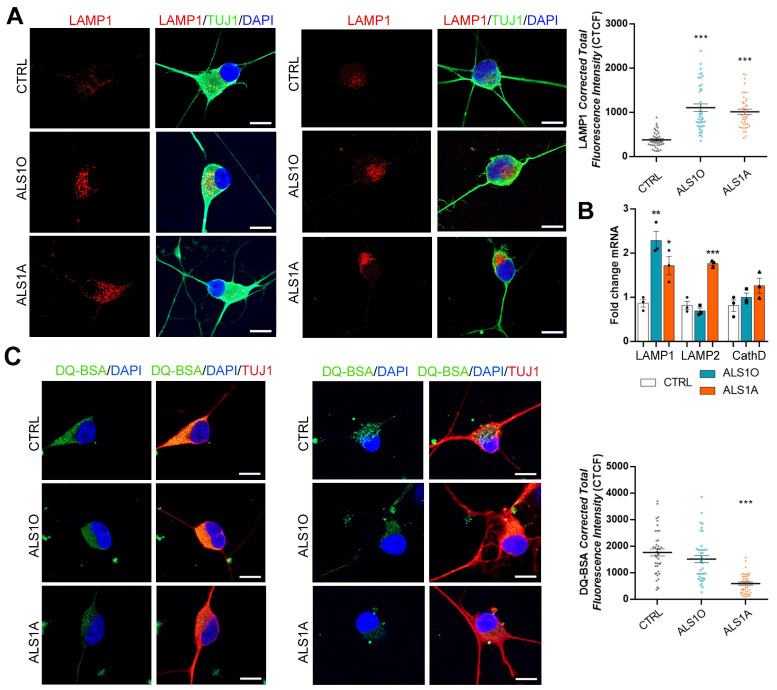
The G376D mutation in TDP-43 affects lysosomal activity in motor neurons in the late stages of ALS disease. (**A**) iPSC-derived motor neurons were fixed and immunolabeled with LAMP1 antibody, followed by Alexa568-conjugated secondary antibody. Nuclei were stained with DAPI. Bar = 10 µM. The Corrected Total Cell Fluorescence (CTCF) was calculated, and statistical analysis was performed using one-way ANOVA, followed by Dunnett’s test for multiple comparisons. (**B**) LAMP1, LAMP2, and Cathepsin D mRNA levels were quantified, using Rplp0 as a control, by real-time PCR in control and ALS iPSCs. Statistical analysis was performed using one-way ANOVA, followed by Dunnett’s test for multiple comparisons. * = *p* < 0.05; ** = *p* < 0.01; *** = *p* < 0.001. (**C**) iPSC-derived motor neurons were treated with 50 µg/mL DQ-BSA for 24 h, and then fixed and immunolabeled with TUJ1 antibody, followed by Alexa568-conjugated secondary antibody. Nuclei were stained with DAPI. Bar = 10 µM. The Corrected Total Cell Fluorescence (CTCF) was calculated, and statistical analysis was performed using one-way ANOVA, followed by Dunnett’s test for multiple comparisons. * = *p* < 0.05; ** = *p* < 0.01; *** = *p* < 0.001.

As expected, iPSC-derived motor neurons showed expression of the neuronal marker TUJ1, confirming that they were correctly differentiated (Figure 8A,C).

Then, we investigated LAMP1 abundance by immunofluorescence analysis, and observed that it was significantly increased in ALS motor neurons compared to the control, confirming the data obtained in fibroblasts (Figure 8A).

Moreover, we decided to evaluate the mRNA levels of some lysosomal markers in iPSCs. ALS iPSCs showed higher levels of LAMP1 transcript at both early and advanced stages of the disease, while LAMP2 mRNA was more abundant only at the late stages. On the contrary, Cathepsin D mRNA levels were unchanged in ALS iPSCs compared to control cells (Figure 8B).

Subsequently, we measured lysosomal activity by incubating iPSC-derived neurons with DQ-BSA and evaluating the fluorescence intensity. We observed that iPSC-derived ALS1A motor neurons had reduced lysosomal activity, as DQ-BSA fluorescence intensity was significantly reduced. In contrast, in iPSC-derived ALS1O motor neurons, the lysosomal activity, measured as the intensity of DQ-BSA staining, was comparable to that of control cells (Figure 8C).

These data indicate that the G376D mutation in TDP-43 also affects lysosomal functionality in iPSC-derived motor neurons, confirming the data obtained for fibroblasts, and they indicate that this pathological phenotype worsens with the progression of the disease, since the ALS1O cells were similar to the control cells.

## 3. Discussion

In this study, we demonstrated that the presence of the G376D mutation in TDP-43 is associated with an increased abundance of late endosomes and lysosomes. Indeed, late endocytic and lysosomal markers were more abundant in ALS cells, suggesting that the number of lysosomes was increased in TDP-43^G376D^-expressing cells, although they displayed reduced lysosomal activity. These data are consistent with several reports indicating that dysregulation in the degradative endocytic route contributes to the onset and progression of neurodegenerative diseases, such as Alzheimer’s disease, frontotemporal dementia, and ALS [21,39].

Genetic mutations in *TARDBP* were linked to ALS for the first time in 2008 [40]. Most of the identified mutations are localized in the C-terminal region of TDP-43, making this protein more prone to generating cytoplasmic aggregates, and reducing its nuclear localization [3]. Mutations in other ALS-related genes, such as C9orf72, are also characterized by TDP-43 accumulation in the cytosol, affecting its ability to shuttle between the cytoplasm and the nucleus [21]. This alters TDP-43′s pleiotropic functions, leading to changes in its splicing profile, impairment of mitochondria, dysregulation of metal ion homeostasis, impaired nucleosomes, dysregulation of autophagy, and disruption of lysosomal dynamics [3,41,42,43,44].

Besides being involved in ALS, TDP-43 is important for the lysosomal pathway. Indeed, the loss of TDP-43 induces nuclear translocation of TFEB, a master regulator of lysosomal biogenesis, and increases lysosomal biogenesis [20,45]. This is consistent with the data presented in this paper. Indeed, it has been previously demonstrated that the G376D mutation leads to TDP-43 mislocalization, with the formation of cytoplasmic aggregates, and diminished localization of the protein in the nucleus [23,24]. This results in the alteration of the nuclear functions of TDP-43. Here, we demonstrated that the G376D mutation induced nuclear translocation of TFEB, thus increasing lysosomal biogenesis, following the observation of Xia et al. [20]. Indeed, we showed that RAB7A, LAMP1, LAMP2, CD63, and the V1G1 subunit of vacuolar ATPase were increased in ALS cells compared to control cells. Thus, the observed accumulation of lysosomes could result from the loss of TDP-43 nuclear functions. Interestingly, CD63 also has a role in exosome biogenesis, since its knockout leads to reduced production of small vesicles [46]. Moreover, extracellular vesicles (EVs) derived from ALS cellular and animal models contain protein aggregates, and this could represent a mechanism for the propagation of the proteinopathy [47]. Therefore, the increase in CD63 levels could suggest the presence of more lysosomes, but also the generation of more EVs that could transfer TDP-43 aggregates between cells. This aspect should be investigated shortly.

Interestingly, in cells from a patient at the time of diagnosis (ALS1O, both skin fibroblast and neurons), lysosomal markers were increased. However, lysosomal functionality was still unaffected in the same patient, as well as in another patient at the late stage of the disease (ALS1A and ALS2A), and lysosomal activity was compromised. These data suggest that initial impairment of lysosomal function due to the onset of the disease causes the activation of TFEB that is translocated in the nucleus, promoting biogenesis of lysosomes to restore lysosomal activity. Thus, in the early stages of the disease, the increased biogenesis of lysosomes and, consequently, the increase in the number of lysosomes can counteract the effects of the mutation on lysosomal functionality. However, this is not sufficient when the disease progresses, indicating that lysosomal alterations worsen with the progression of the disease, and are no longer correctable by TFEB action. Indeed, the result is the accumulation of dysfunctional lysosomes, since these organelles showed impaired degradative activity and acidification. Since lysosomes are important for eliminating TDP-43 aggregates, the progressive lysosomal dysfunction results in severe protein aggregation in the late stages of the disease, but not in the early stages, as demonstrated in our previous work [24].

Moreover, we previously observed that both overexpression and silencing of V1G1 caused the accumulation of immature forms of Cathepsin D, demonstrating that V1G1 is important to ensure the pump’s proper activity [30]. Therefore, the altered amount of V1G1 present in ALS cells suggests that the acidification of the lysosomal lumen could be affected in cells carrying the TDP-43^G376D^ mutation, thus affecting lysosomal function. To assess this aspect, we used Lysosensor DND-160, and this experiment allowed us to establish that ALS fibroblasts are characterized by impaired acidification. In these cells, we also observed defects in Cathepsin D maturation and activity, concurrently with an increased abundance of V1G1 and impaired acidification. Indeed, Cathepsin D activity depends on low pH inside the endo/lysosomal compartments, which allows the degradation of materials transported into the lysosomes [48]. Therefore, defective lysosomal acidification led to the reduced Cathepsin D activity observed in fibroblasts carrying the TDP-43^G376D^ mutation.

Interestingly, Cathepsin D is the only aspartic-type protease expressed in all cells of the body, and its levels are particularly high in neuronal cells. Therefore, the alteration of cathepsin D activity is detrimental for these cells, leading to the onset and progression of several neurodegenerative diseases [49].

The upregulation of TFEB activity might represent a mechanism to compensate for lysosomal dysfunction. This mechanism is a feature observed in other neurodegenerative diseases. For instance, in a mouse model of Alzheimer’s disease, protein accumulation in lysosomes resulted in lysosomal stress that led to TFEB activation, representing a line of defense that is, however, quickly overwhelmed, resulting in the progression of the disease [50]. Therefore, a vicious circle could be established. Indeed, protein accumulation leads to TFEB activation and increased lysosomal biogenesis. However, although these organelles are abundant, they do not work properly, as demonstrated by impaired lysosomal degradative activity and acidification, leading to protein accumulation. Our experiments also indicated that increased TFEB nuclear translocation was not dependent on reduced phosphorylation from mTORC1, but was instead due to reduced AKT activation. Interestingly, similarly to what we observed, in a mouse model of ALS carrying the SOD1^G93A^ mutation, AKT phosphorylation was reduced [51]. Also, AKT inactivation characterizes the skeletal muscle of ALS animals, responsible for iron accumulation and disease progression [51].

The accumulation of TFEB in the Golgi apparatus in ALS fibroblasts endogenously expressing the TDP-43^G376D^ mutant protein is worth noting. A similar altered subcellular localization was observed in dopaminergic neurons in the substantia nigra pars compacta of subjects with sporadic Parkinson’s Disease and Dementia with Lewy bodies [52]. Even though the mechanisms behind TFEB accumulation in the Golgi apparatus are still unknown, the mislocalization of TFEB in the Golgi apparatus could be a common trait of different neurodegenerative diseases.

Importantly, we were also able to demonstrate the reduction of lysosomal activity in iPSC-derived motor neurons obtained from patients carrying the TDP-43^G376D^ mutation, strengthening the evidence that lysosomal dysfunction caused by this mutation is an important pathogenic mechanism leading to neurodegeneration.

## 4. Materials and Methods

### 4.1. Antibodies and Reagents

The primary antibodies used in this study were the following: anti-LAMP1 (for WB 1:4000; ab24170), anti-histone H3 (1:5000; ab1791), anti-V1G1 (1:1000; ab15853), anti-Giantin (1:200; ab37266) from Abcam (Cambridge, UK), anti-GAPDH (1:2000; sc-25778), anti-β-actin (1:5000; sc-47778), anti-pAkt (1:500; sc-514032), anti-RAB7A (for WB 1:500; sc-376362), anti Hsp90 (sc-13119, 1:5000), anti-Cathepsin D (sc-6486; 1500), anti-CD63 (sc-5275; 1:200) from Santa Cruz Biotechnologies (Santa Cruz, CA, USA), anti-TDP-43 (1:3000, 10782-2-AP) from Proteintech (Rosemont, IL, USA), anti-Akt (1:1000; #4691), anti-Raptor (1:1000; #2280), anti-mTOR (1:1000; #2983) from Cell Signaling Technology (Danvers, MA, USA), anti-LAMP1 (for IF 1:250; H4A3) and anti-LAMP2 (1:5000; H4B4) deposited into the Developmental Studies Hybridoma Bank (Iowa City, IA, USA), anti-TFEB (for WB 1:1000; A303-673A) from Bethyl Laboratories (Montgomery, TX, USA), anti-Vinculin (1:5000; V9131), anti-TUJ1 (1:1000; T2200), and anti-TFEB (for IF 1:100; HPA049532) from Sigma Aldrich (St. Louis, MO, USA).

The HRP-conjugated secondary antibodies used for Western blot analysis were the following: anti-rabbit (#1706515), anti-goat (#1721034), and anti-mouse (#1706516), all purchased from Bio-Rad (Hercules, CA, USA), and anti-chicken (AP194P) from Merck Millipore (Burlington, MS, USA). They were all used at 1:5000 dilution. The secondary antibodies used for immunofluorescence analysis were the following: anti-mouse Alexa Fluor 488-conjugated (A21202), anti-rabbit Alexa Fluor 488-conjugated (A21206), anti-mouse Alexa Fluor 555-conjugated (A31570), and anti-rabbit Alexa Fluor 555-conjugated (A31572), all from Life Technologies (Carlsbad, CA, USA) and diluted by 1:400.

### 4.2. Cell Lines

HeLa cells (CVCL_0030) were grown in Dulbecco’s Modified Eagle Medium (DMEM) supplemented with 10% FBS (Fetal Bovine Serum), 2mM L-Glutamine, 100 U/mL penicillin, and 10 mg/mL streptomycin.

Dermal fibroblasts were derived from two healthy subjects (control fibroblasts) and two patients carrying the TDP-43^G376D^ mutation (ALS1A and ALS2A). ALS1O fibroblasts were derived from patient 1 at the time of diagnosis. Fibroblasts were obtained, after informed consent, by skin biopsies, and cultured under protocols approved by the Università Cattolica del Sacro Cuore Ethics Committee (Protocol P/740/CE/2012) and the Palermo 1 Ethics Committee (Protocols 7/2017 and 4/2019). These cells were grown in DMEM supplemented with 15% FBS, 2 mM L-Glutamine, 100 U/mL penicillin, and 10 mg/mL streptomycin. DMEM (ECM0728L) and FBS (ECS5000L) were obtained from Euroclone (Pero, Italy), and L-Glutamine (25030081), penicillin, and streptomycin (15140-122) were obtained from Gibco (Amarillo, TX, USA). All cell lines were incubated in a 5% CO_2_ incubator at 37 °C, and checked to ensure they were contamination-free.

Motor neurons were obtained from iPSCs nucleofected with epB-Bsd-TT-NIL, as described by Garone et al. [53]. NIL-inducible iPSC lines were plated on a 6-well plate coated with Matrigel at a density of 62,500 cells/cm^2^, and grown following the protocol. On day 5 of differentiation, cells were dissociated and plated on cultrex-coated glass coverslips at a density of 30,000 cells/coverslip, and differentiated into spinal motor neurons up to day 14.

### 4.3. Transfection

The plasmid coding for EGFP-TDP-43^wt^ has been previously described [54]. From this construct, we obtained a plasmid encoding EGFP-TDP-43^G376D^ by site-directed mutagenesis, using the PfuTurbo DNA polymerase (600252-52, Agilent Technologies, Santa Clara, CA, USA), following the manufacturer’s instructions, using the following primers: 5′-GGAAATAACTCTTATATAGTGACTCTAATTCTGGTGCAGC-3′ and 5′-GCTGCACCAGAATTAGAGTCACTATAAGAGTTATTTCC-3′. The empty vector p-EGFP-C_1_ was used as a control in the transfection experiments.

HeLa cells were transfected using Metafectene Pro (T040, Biontex, Martinsried, Germany), according to the manufacturer’s instructions, and the cells were processed 48 h after transfection.

### 4.4. Western Blotting

Cells were lysed in Laemmli buffer [(100 mM Tris-HCl, pH 6.8, 4% (*w*/*v*) SDS, 0.2% (*w*/*v*) bromophenol blue, 20% glycerol, and 200 mM DTT (dithiothreitol)] and processed for Western blot analysis as previously described [55,56]. Briefly, after SDS-PAGE, proteins were transferred on PVDF (polyvinylidene) (IPVH00010, Merck-Millipore, Burlington, MS, USA) or nitrocellulose (1620112, Bio-Rad, Hercules, CA, USA) membranes. Membranes were then blocked with 5% milk in PBS (phosphate-buffered saline) 1X (137 mM NaCl, 2.7 mM KCl, 10 mM Na_2_HPO_4_, 1.8 mM KH_2_PO_4_) for 30 min, and then incubated with the appropriate primary antibodies overnight at 4 °C, and with the appropriate secondary HRP-conjugated antibodies for 1 h at room temperature, all diluted in 1% milk in PBS 1X. Clarity (170-5061) or Clarity Max (170-5062) kits (Bio-Rad, Hercules, CA, USA) were used for the chemiluminescence reaction, and the signal was captured by ChemiDoc MP Imaging Systems (Bio-Rad). Densitometric analysis was performed using Image Lab software 6.1 (Bio-Rad).

### 4.5. Nuclear and Cytoplasmic Fractionation

Cells were lysed in lysis buffer (50 mM Tris pH 7.5, 0.1% Triton X-100, 137.5 mM NaCl, 10% glycerol, 5 mM EDTA) as previously described [24]. The nuclear and cytoplasmic fractions obtained were then processed by Western blot analysis.

### 4.6. Immunofluorescence and Confocal Microscopy

Cells grown on 11 mm coverslips were permeabilized with 0.25% saponin in PIPES-EGTA (80 mM PIPES (pH 6.8), 5 mM EGTA, and 1 mM MgCl_2_) for 2 min at room temperature, and then fixed with 3% paraformaldehyde (PFA) for 20 min at room temperature. Motor neurons were fixed with 4% paraformaldehyde (PFA) for 15 min at room temperature, and permeabilized with 0.1% Triton in PBS. Primary and secondary antibodies were diluted in 0.1% Saponin in PBS. Nuclei were stained with DAPI for 5 min. The cells were washed several times, and the coverslips were mounted with Mowiol. Images were acquired with an LSM900 confocal scanning microscope equipped with a 63x oil-immersive objective (Zeiss, Oberkochen, Germany).

For TFEB and mTOR immunofluorescence, cells were treated as previously reported [20]. In brief, cells were fixed with 3% paraformaldehyde at room temperature for 20 min, permeabilized with 0.25% Triton X-100 in PBS for 10 min, and blocked with 5% BSA (Bovine Serum Albumin)-0.3% Triton X-100 in PBS for 30 min. Cells were then incubated with primary antibodies for 6 h and with secondary antibodies for 2 h.

For live imaging, we seeded cells on an 8-well μ-slide (Ibidi GmBh, Martinsried, Germany). The next day, we incubated cells with 1 µM Lysosensor Yellow/Blue DND-160 (L7545, ThermoFisher Scientific, Waltham, MA, USA) for 5 min at 37 °C. This probe can detect organelles with a pH range of 3.5–6.0. After this incubation, the cells were washed three times with PBS, L-15 medium (Leibowitz medium without phenol red, 21083027, Gibco, ThermoFisher, Waltham, MA, USA) was added, and images were acquired using an LSM900 confocal scanning microscope equipped with a 63× oil-immersive objective (Zeiss, Oberkochen, Germany).

### 4.7. DQ-BSA (Self-Quenched BODIPY Dye Conjugated of Bovine Serum Albumin) Assay and Live Cell Staining

The cells previously seeded on the 11 mm glass coverslips were incubated in the presence of 10 µg/mL Red DQ-BSA (D12051, ThermoFisher Scientific, Waltham, MA, USA) for 48 h, at 37°C, in a 5% CO_2_ cell culture incubator in the case of fibroblasts, and HeLa cells were incubated with 25 µg/mL Red DQ-BSA for 24 h, while neurons were incubated with 50 µg/mL Green DQ-BSA (D12050, ThermoFisher Scientific, Waltham, MA, USA) for 24 h under the same conditions. Cells were fixed with 3% PFA for 20 min, and nuclei were stained with DAPI for 5 min. Coverslips were mounted with Mowiol, and images were acquired with an LSM700 confocal laser scanning microscope equipped with a 63X objective (Zeiss, Oberkochen, Germany).

### 4.8. EGF Degradation Assay

Cells were incubated overnight with a starvation medium (0.5% BSA, 20 mm HEPES pH 7.3 in DMEM). The next morning, cells were incubated with 0.8 mg/mL rhodamine-labeled EGF (E3481, ThermoFisher Scientific, Waltham, MA, USA) for 1 h at 4 °C in a starvation medium. Then, the cells were washed three times with starvation medium, a complete DMEM was added, and the cells were incubated in a 5% CO_2_ incubator at 37 °C. The cells were fixed and processed for confocal microscopy.

### 4.9. Cathepsin D Activity

Cathepsin D activity was evaluated using the Cathepsin D Activity Fluorometric Assay (K143-100, BioVision, Milpitas, CA, USA), following the manufacturer’s instructions. Briefly, 20,000 control and ALS fibroblasts were collected and lysed. The lysates were incubated for 1 h at 37 °C with cathepsin-D substrate sequence GKPILFFRLK(Dnp)-D-R-NH2 labeled with fluorescent MCA (7-methoxycoumarin-4-acetic acid), and then the fluorescence was read at Ex/Em = 328/460 nm.

### 4.10. Real-Time PCR

hiPSC pellets were processed with Trizol reagent (15596026, ThermoFisher Scientific, Waltham, MA, USA). Extracted RNAs were quantified with a Qubit 3.0 Fluorometer, and the quality was validated with the Nanodrop 1000. The RNAs were digested with DNase I, before proceeding with reverse transcription using the High Capacity cDNA Reverse Transcription kit (4374967, ThermoFisher Scientific, Waltham, MA, USA). PCR reactions were prepared with the SYBR^TM^ Green PCR Master Mix (4367659, Applied Biosystem, Waltham, MA, USA), and run in a C1000 Touch Thermal Cycler (Bio-Rad, Hercules, CA, USA). The thermal profile used for real-time PCR was as follows: 1 cycle of 2 min at 50 °C; 1 cycle of 10 min at 95 °C; 40 cycles of 15 s at 95 °C, 1 min at 55 °C; 1 cycle of 15 s at 95 °C and 15 s at 60 °C. Quantification was performed as previously described [57].

The primers used are listed below:

Cathepsin D: forward: 5′-CAGAAGCTGGTGGACCAGAAC-3′,

reverse: 5′-TGCGGGTGACATTCAGGTAG-3′.

LAMP1: forward: 5′-ACGTTACAGCGTCCAGCTCAT-3′,

reverse: 5′-TCTTTGGAGCTCGCATTGG-3′.

LAMP2: forward: 5′-TGCTGGCTACCATGGGGCTG-3′,

reverse: 5′-GCAGCTGCCTGTGGAGTGAGT-3′.

Rplp0: forward: 5′-TCGACAATGGCAGCATCTAC-3′.

reverse: 5′-ATCCGTCTCCACAGACAAGG-3′.

All the primers were purchased from Eurofins Genomics (Ebersberg, Germany).

### 4.11. Senescence-Associated Beta-Galactosidase (SA-βgal) Activity Assay

Cells were seeded in a 6-well plate, and the next day, they were washed with PBS and fixed in 2% formaldehyde and 0.2% glutaraldehyde in PBS for 5 min. After three washes in PBS, the cells were stained with 40 mM citric acid/Na phosphate buffer at pH 6, 1 mg/mL X-gal, 5 mM K_4_[Fe(CN)_6_]_3_H_2_O, 5 mM K_3_[Fe(CN)_6_], 150 mM NaCl, and 1 mM MgCl_2_ in ddH_2_O, at 37 °C, overnight. Finally, the cells were washed three times with PBS and one time with methanol. Images were acquired using the EVOS FL Auto Cell Imaging System (ThermoFisher Scientific, Waltham, MA, USA).

### 4.12. Statistical Analysis

All experiments were performed at least three times. The data were statistically analyzed using one-way ANOVA, followed by Dunnett’s test for multiple comparisons or Student’s t-test (* = *p*  <  0.05, ** = *p*  <  0.01 and *** = *p*  <  0.001). The graphs represent the mean value ±  SEM (Standard Error Mean).

The fluorescence intensity was determined by ImageJ Software 1.54d, and it was evaluated by quantifying the Corrected Total Cell Fluorescence (CTCF), as previously described [58]. The colocalization rate was determined by the Zen 2011 software black edition 3.3 (Carl Zeiss, Oberkochen, Germany) as the weighted colocalization coefficient of mTOR and LAMP1, as previously described [59].

## 5. Conclusions

In this paper, we evaluated the effect of the ALS-causing mutation TDP-43^G376D^ on the lysosomal pathway. We demonstrated that this mutation is associated with an increase in lysosome number and reduced lysosomal activity, as demonstrated by reduced DQ-BSA fluorescence, but also with impaired EGF degradation, impaired cathepsin D activity and maturation, and altered acidification. ALS cells tend to compensate for lysosomal dysfunction by increasing TFEB nuclear translocation, and this mechanism is dependent on AKT-reduced activation. However, this line of defense is quickly overwhelmed, resulting in the accumulation of dysfunctional lysosomes, protein aggregation, and the progression of the disease. Our data identified several proteins important for the lysosomal pathway whose expression or activity changed in ALS cells, such as cathepsin D. Interestingly, in a mouse model of Alzheimer’s disease, lysosomal pH was restored using cilostazol, re-establishing Cathepsin D activity, and reducing aberrant protein aggregates [60]. Therefore, lysosomal proteins could become targets for understanding and treating this currently incurable disease.

## Data Availability

The original contributions presented in this study are included in the results. Further inquiries can be directed at the corresponding authors.
